# Supplementary material for: An active allosteric mechanism in ASAP1-mediated Arf1 GTP hydrolysis redefines PH domain function
Source: Nat Commun. 2025 Sep 30;16:8701. doi: 10.1038/s41467-025-63764-w (PMC12485133; doi:10.1038/s41467-025-63764-w)
Supplement: Supplementary file 1 — Supplementary Information [file 41467_2025_63764_MOESM1_ESM.pdf]

FIG. SI1

A

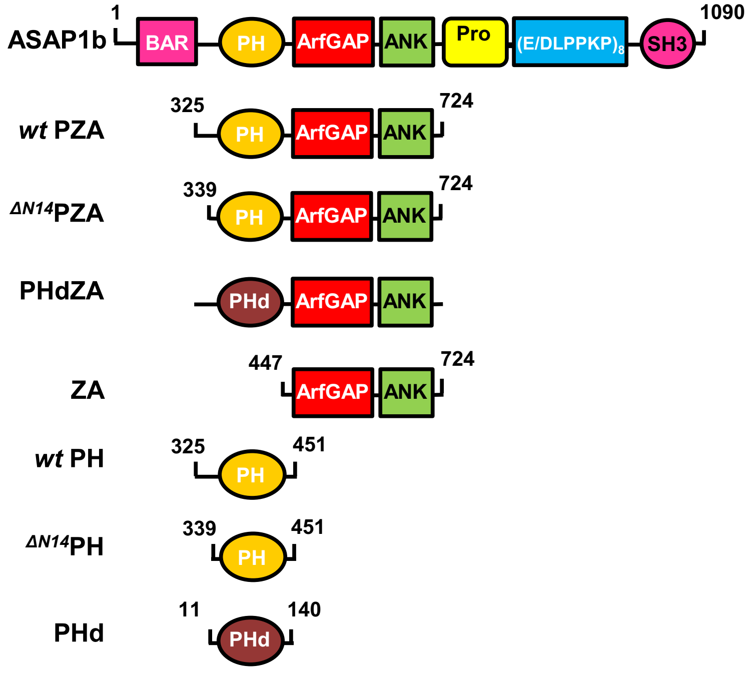

ASAP1b
1
1090
BAR
PH
ArfGAP
ANK
Pro
(E/DLPPKP)8
SH3
wt PZA
325
724
ΔN14PZA
339
724
PHdZA
PHd
ZA
447
724
wt PH
325
451
ΔN14PH
339
451
PHd
11
140

B

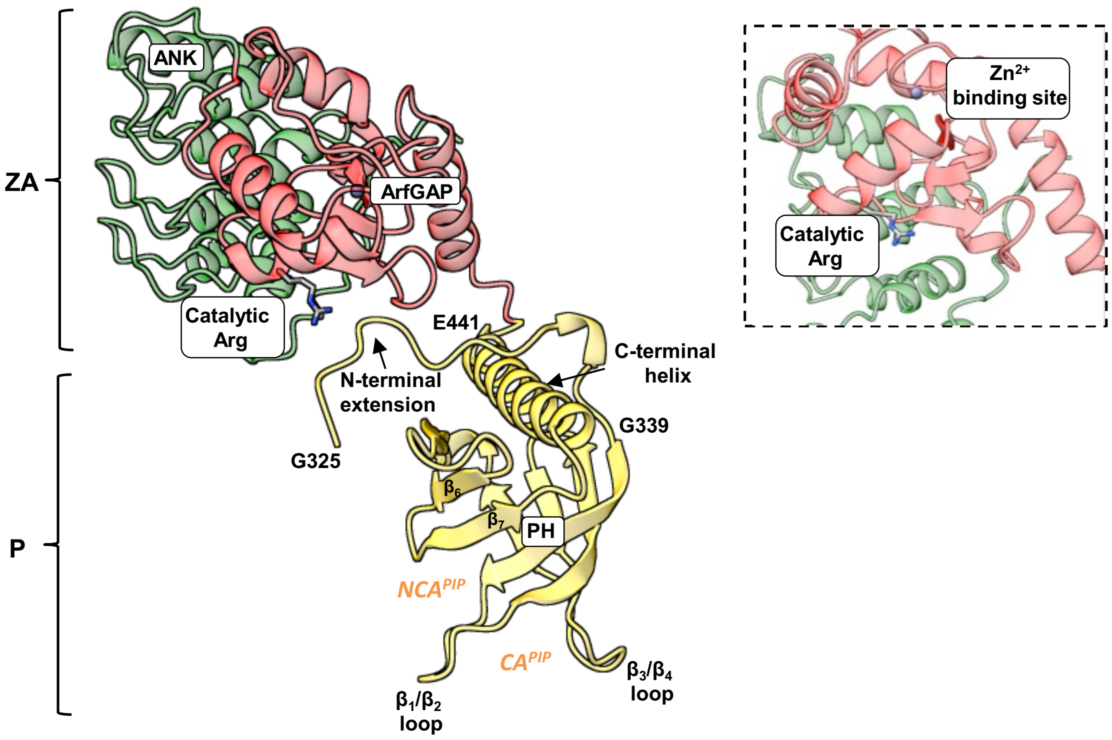

ZA
P
ANK
ArfGAP
Catalytic Arg
E441
N-terminal extension
C-terminal helix
G339
G325
β6
β7
PH
NCAPIP
CAPIP
β1/β2 loop
β3/β4 loop
Zn2+ binding site
Catalytic Arg

Fig. SI1. (A) Schematic of recombinant proteins used in this paper. The domain structure of ASAP1 is shown in the schematic at top. Abbreviations: BAR, Bin/amphiphysin/RVS; PH, pleckstrin homology; Arf GAP, Arf GTPase-activating protein; ANK, ankyrin repeat; Pro-Rich, proline-rich; (E/DLPPKP)8, tandem repeats of E/DLPPKP; SH3, Src homology 3. Recombinant proteins used in the studies are shown below the schematic of full-length ASAP1. The acronyms for the proteins include "P" for the PH domain, "Z" for the Arf GAP domain, which is a zinc-binding motif, "A" for the ankyrin repeat and "PHd" for the PH domain of phospholipase Cδ1. PHdZA is a chimeric protein consisting of residues 11 to 140 of PLCδ1 and residues 441 to 724 of ASAP1 **(B)** Ribbon representation of the AlphaFold structure of *wt* PZA. The PH (in yellow) and ZA (in red/green) domains behaves like "beads-on-a-string". PH domains are defined by a structural fold of 7 β-strands arranged as a sandwich and capped by a C-terminal amphipathic α-helix with loops of different lengths connecting the β-strands. For visual guidance, β strands 5-7, as well as loops linking the β strands and C-terminal helix are labeled. Approximate sites of $PI(4,5)P_2$ interaction are labeled CA (for canonical sites) and NCA (for noncanonical site). Inset: Ribbon representation of part of the ZA domain, highlighting the catalytic Arginine and the Zn^{2+} binding site.

FIG. SI2

A

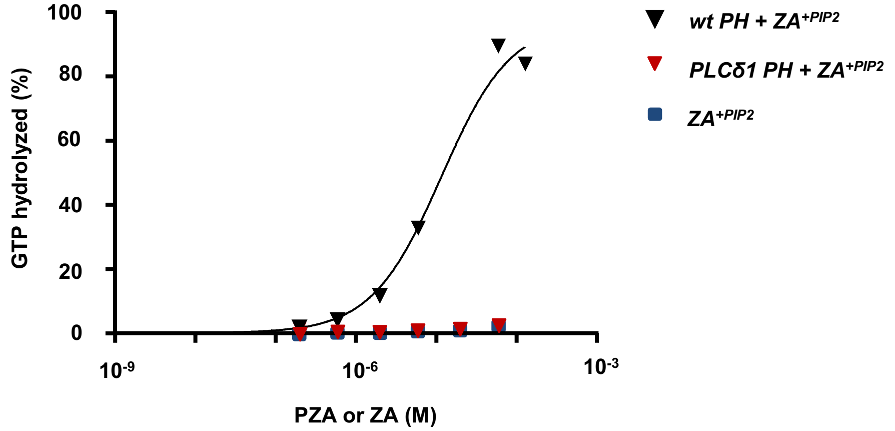

B

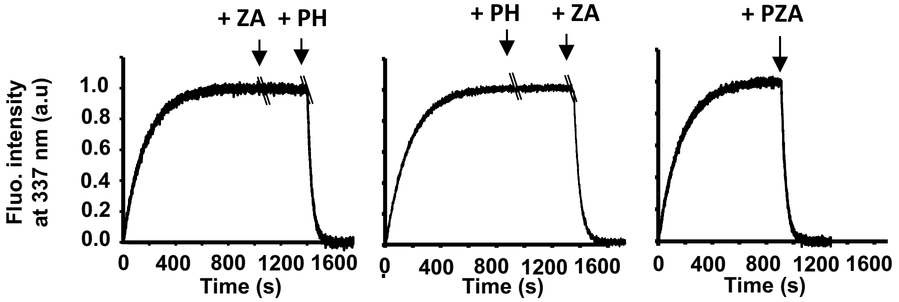

Fig. SI2. (A) Comparison of GAP activity using Arf1 as substrate in the presence of PI(4,5)P_2 containing membranes. *wt* PH + ZA (black ▼), PHd (PLCδ1 PH) + ZA (maroon ▼) or ZA (blue ■) was titrated into a GAP reaction containing 1 µM full-length Arf1 and LUVs at a total phospholipid exposed concentration of 0.25 mM containing 1% mol PI(4,5)P_2. **(B)** Representative tryptophan fluorescence kinetics trace of GTP hydrolysis of myr-Arf1 after GDP/GTP exchange triggered by the addition of PH and ArfGAP-Ankyrin repeat domains either isolated or in tandem. **Left:** Isolated ZA domain (25 µM) was added to myrArf1·GTP (5µM) for ~ 600 s before addition of *wt* PH domain (5 µM). **Middle:** Isolated *wt* PH domain (5 µM) was added to myrArf1·GTP (5µM) bound to ND for ~ 500 s before addition of ZA domain (25 µM). **Right:** *wt* PZA domain

(10 nM) was added to myrArf1·GTP (5 μM) when GDP/GTP exchange was complete (800 s). Nucleotide exchange Arf·GDP (5 μM) was triggered by the addition of 2 mM EDTA in the presence of 20 μM GTP, at 22 °C in the presence of ND containing 0.5 mM of accessible lipids.

FIG. SI3

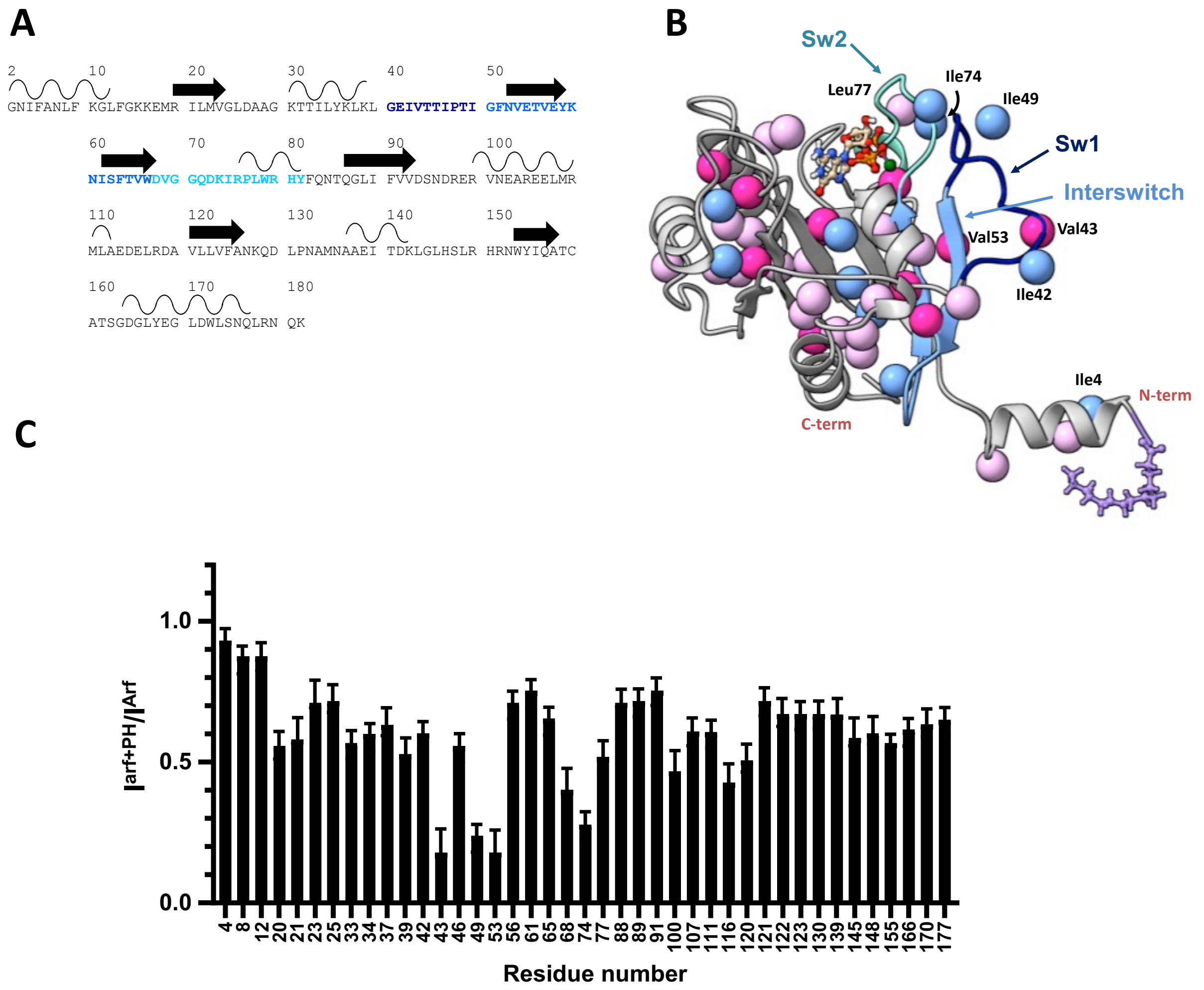

Fig. SI3. (A) Amino acid sequence and secondary structure elements of Arf1. Switch 1 (dark blue), switch 2 (cyan) and the IS (corn blue) are highlighted. **(B)** Homology model of human myr-Arf1, generated based on yeast Arf1 (PDB:2KSQ) using MODELLER, is shown in grey ribbon format. Switch 1 (dark blue), switch 2 (cyan) and the interswitch (light blue) and methyl-containing residues are highlighted: 11 isoleucines (blue), 22 leucines (light pink), and 11 valines (dark pink). The myristoyl chain (purple) is shown as a ball and stick representation. Residues 2–13 form the N-terminal helix (embedded in the nanodisc), and residues 17–181 constitute the G-domain (solvent-exposed). For leucine and valine residues, only the Pro-S methyl carbons are shown.

Images created using Chimera. **(C)** Attenuation of Arf methyl resonances upon complex formation. Ratio of cross peak intensity I^{Arf+PH} / I^{Arf} observed for methyl residues with (I^{Arf+PH}) and without (I^{Arf}) PH domain. When in complex with *wt* ASAP1 PH, loss of rotational freedom leads to an average resonance attenuation of 0.65 ± 0.07. Additional selective resonance attenuation observed for residues 43 and 49 of switch 1, 53 of the interswitch and 68 and 74 of switch 2 reflect residues at the interface.

FIG. SI4

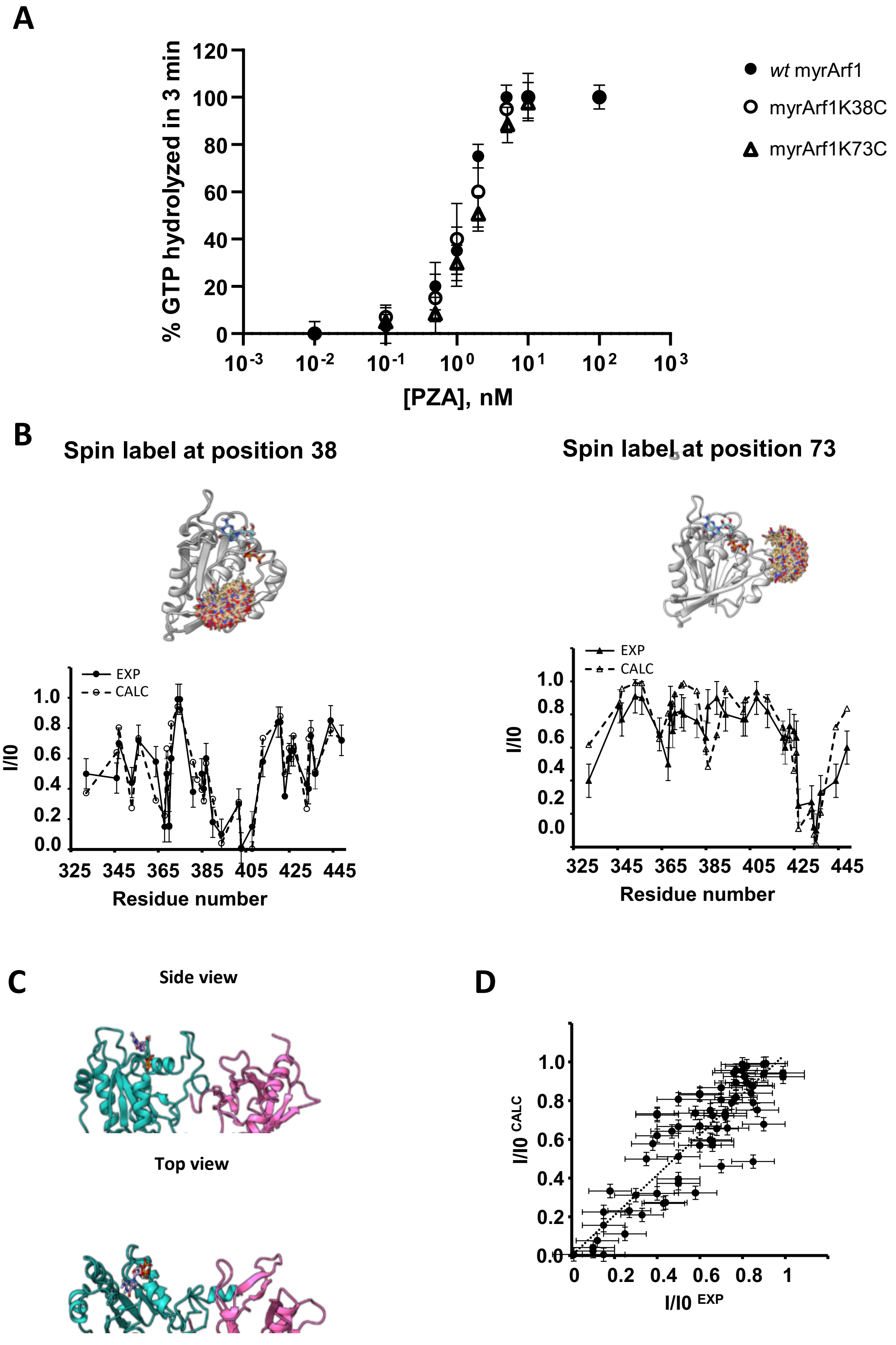
A
% GTP hydrolyzed in 3 min
120
100
80
60
40
20
0
10-3 10-2 10-1 10 0 10 1 10 2 10 3
[PZA], nM
wt myrArf1
myrArf1K38C
myrArf1K73C
B
Spin label at position 38
Spin label at position 73
EXP
CALC
I/I0
1.0
0.8
0.6
0.4
0.2
0.0
325 345 365 385 405 425 445
Residue number
C
Side view
Top view
D
I/I0 CALC
I/I0 EXP
0 0.2 0.4 0.6 0.8 1

Fig. SI4. (A) Functional activity of myrArf1, myrArf1C159AK73C or myrArf1C159AK38C. *wt* PZA was titrated into a reaction containing 5 μM myrArf1·GTP, myrArf1C159AK73C·GTP or myrArf1C159AK38C·GTP as a substrate. The percentage of GTP bound to myr-Arf1 hydrolyzed in 3 min is plotted against *wt* PZA concentration. **(B) left.** Position of MTSL label (brown) on myrArfC159AK38C (in grey). For simplicity, residue 2-17 were omitted. Motion of the label is indicated by the multiple positions depicted. Intermolecular PRE profile measured on ^{13}C methyl labeled ASAP1 PH in the presence of MTSL-tagged myrArf1C159AK38C at the membrane surface. **right.** Position of MTSL label (brown) on myrArfC159AK73C (in grey). For simplicity, Arf is represented without residue 2-17. Intermolecular PRE profile measured on ^{13}C methyl labeled ASAP1 PH in the presence of MTSL-tagged myrArf1C159AK38C at the membrane surface. Two independent experiments were performed. Data are presented as mean values. Error bars were calculated based on the signal-to-noise (S/N) ratio of the spectra as described in Methods. **(C)** Representative snapshot of Arf (cyan) in complex with the ASAP1 PH domain in the $^{\beta}{}_2/^{\beta}{}_3$ orientation (pink) **(D)** Correlation of calculated PRE (y-axis) versus measured PRE (x-axis) corresponding to (**B**). For the measured PRE, error bars were calculated based on the spectral signal-to-noise ratio as described in Methods. For the calculated PRE (y-axis), data are presented as mean values over all MD simulations replicas +/−SD.

FIG. SI5

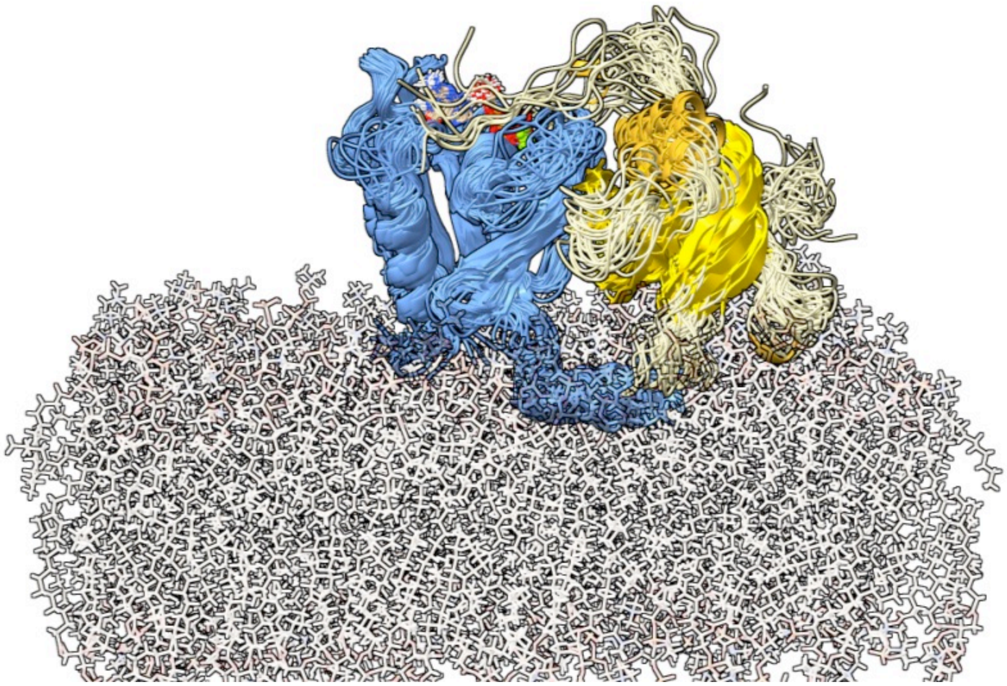

Fig. SI5. A collection of representative conformations of the Arf1:ASAP1 PH complex at the membrane surface obtained from MD simulations. Arf is illustrated in blue, while the ASAP1 PH domain is shown in yellow hues—highlighting the β-sandwich in gold, the C-terminal helix in orange, and the loops and flexible regions in pale yellow.

FIG. SI6

A

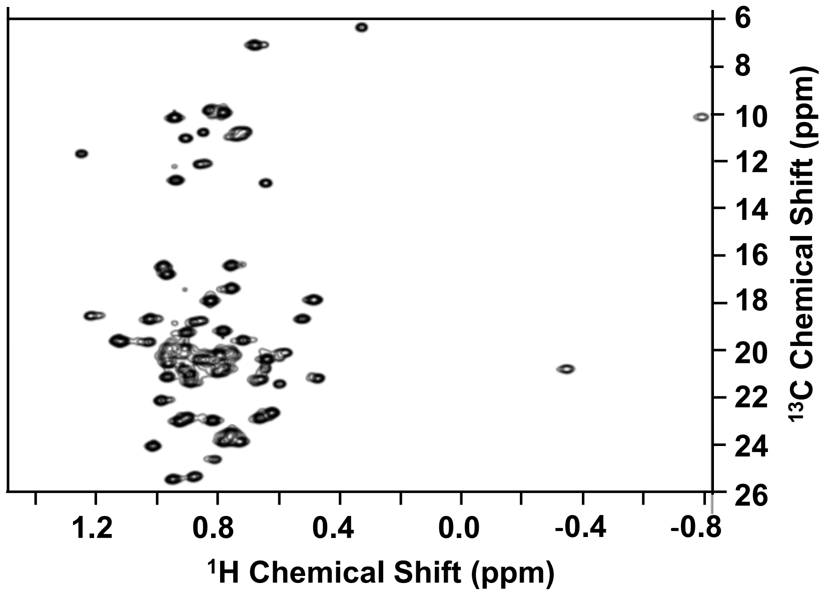

B

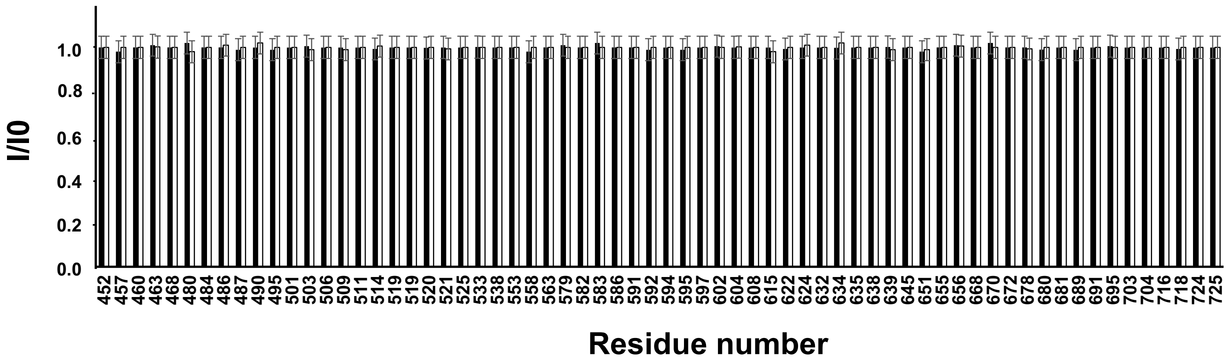

Fig. SI6. (A) ^{1}H-^{13}C HMQC of U-^{2}H,^{15}N and δ1-^{13}C^{1}H-labeled Ile, δ1 -^{13}C^{1}H-labeled Leu and γ1-^{13}C^{1}H-labeled Val ZA domain (100 μM) in the presence of MTSL-tagged myrArf1C159 (100μM) bound to equimolar ratio of ASAP1 PH at the membrane surface. **(B)** Intermolecular PRE ratio

measured on ZA in the presence of MTSL-tagged myrArf1C159 alone (black column) or bound to equimolar ratio of ASAP1 PH (open column) at the membrane surface. No significant PRE could be detected.

FIG. SI7

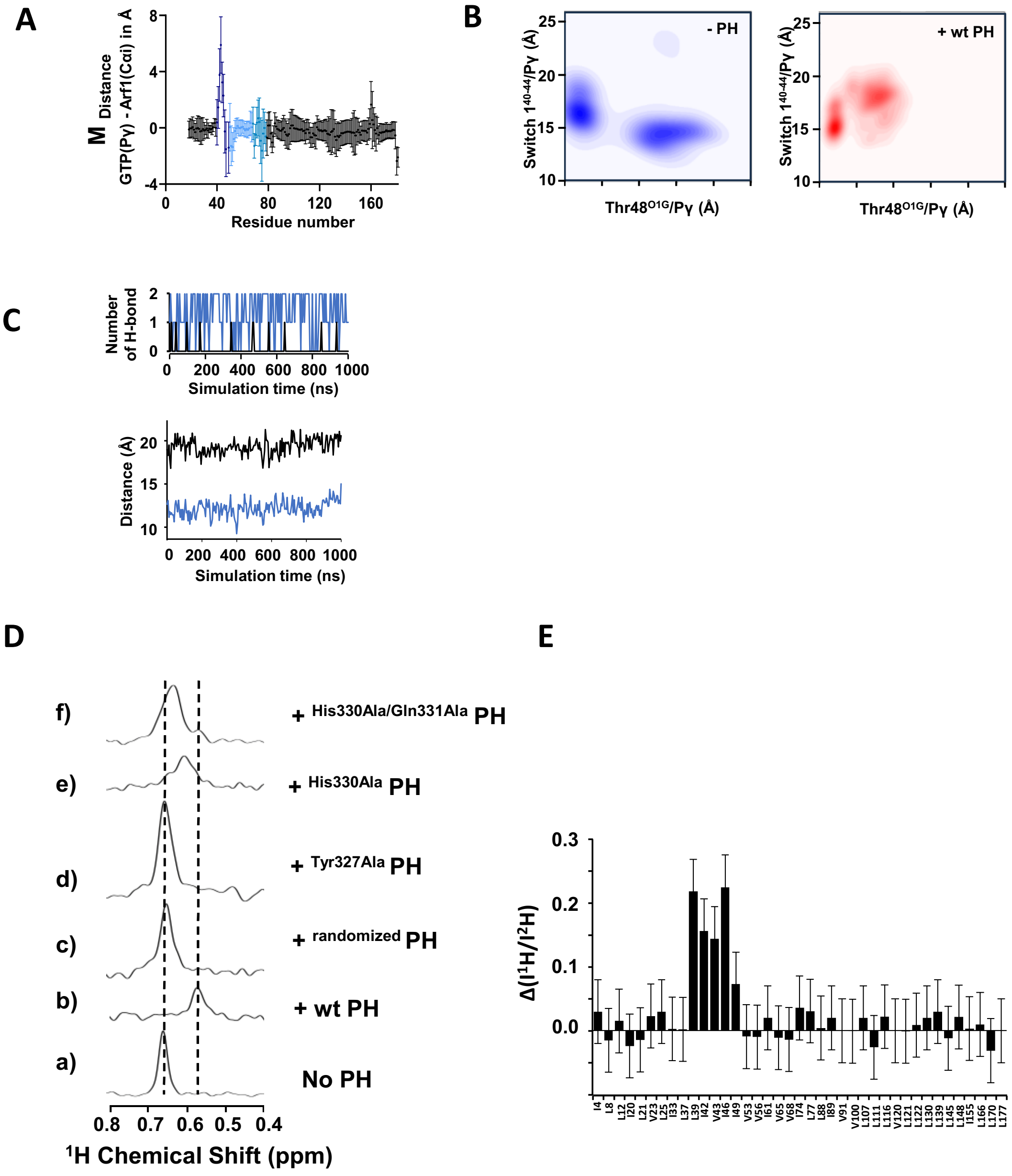

Fig. SI7. (A) Plot of the average distance difference calculated between GTP-Pγ atom of GTP and carbon alpha (CA) of each Arf1 residue calculated as $(d^{GTP\text{-}P\gamma \rightarrow Ca_i}(Arf{:}\mathit{wt}\ PH) - d^{GTP\text{-}P\gamma \rightarrow Ca_i}(Arf))$. Switch 1 (residues 40-49, dark blue), switch 2 (residues 68-78, cyan) and the interswitch (residues 50- 67, light blue) are highlighted. Error bars are calculated as the sum of the SD. **(B)** Correlation between the COM of switch $1^{40\text{-}44}$:GTP-Pγ and Thr48:Mg^{2+} distances for Arf only (blue) and Arf+

wt PH (red) **(C) (top)** Time dependence of the number of H-bonds formed between Glu54 and Thr44 for Arf alone (blue) or for Arf in complex with *wt* PH (black). Only one of the replicas is shown as an example **(bottom).** Time dependence of the distance between the center of mass (COM) of residue 39-45 of switch 1 and GTP-Pγ corresponding to the replica shown. **(D)** Stack of rows extracted from a ^{1}H-^{13}C HMQC experiment along the proton dimension of Val43 (myrArf1) in the absence (a) or in the presence of *wt*-ASAP1 PH (b), $^{\Delta N14}$ASAP1 PH (c) ASAP1 PH with a randomized N terminal extension (GGQLHSYMQ) (d), ^{His330Ala}ASAP1 PH (e) or $^{His330Ala/Gln331Ala}$ ASAP1 PH (f). **(E)** Difference ΔI between the ratio of intensities of Arf1 ^{1}H-^{13}C methyl cross peaks measured in the presence of ^{1}H ASAP1 PH and ^{2}H ASAP1 PH using wt- ASAP1 PH or $^{\Delta N14}$ASAP1 PH plotted against residue number. ΔI is calculated as $(I^{1H\text{-}wt\text{-}PH}/I^{2H\text{-}wt\text{-}PH})/(I^{1H\text{-}\Delta N14\text{-}PH}/I^{2H\text{-}\Delta N14\text{-}PH})$. Error is calculated as the sum of error of each experiment.

FIG. SI8

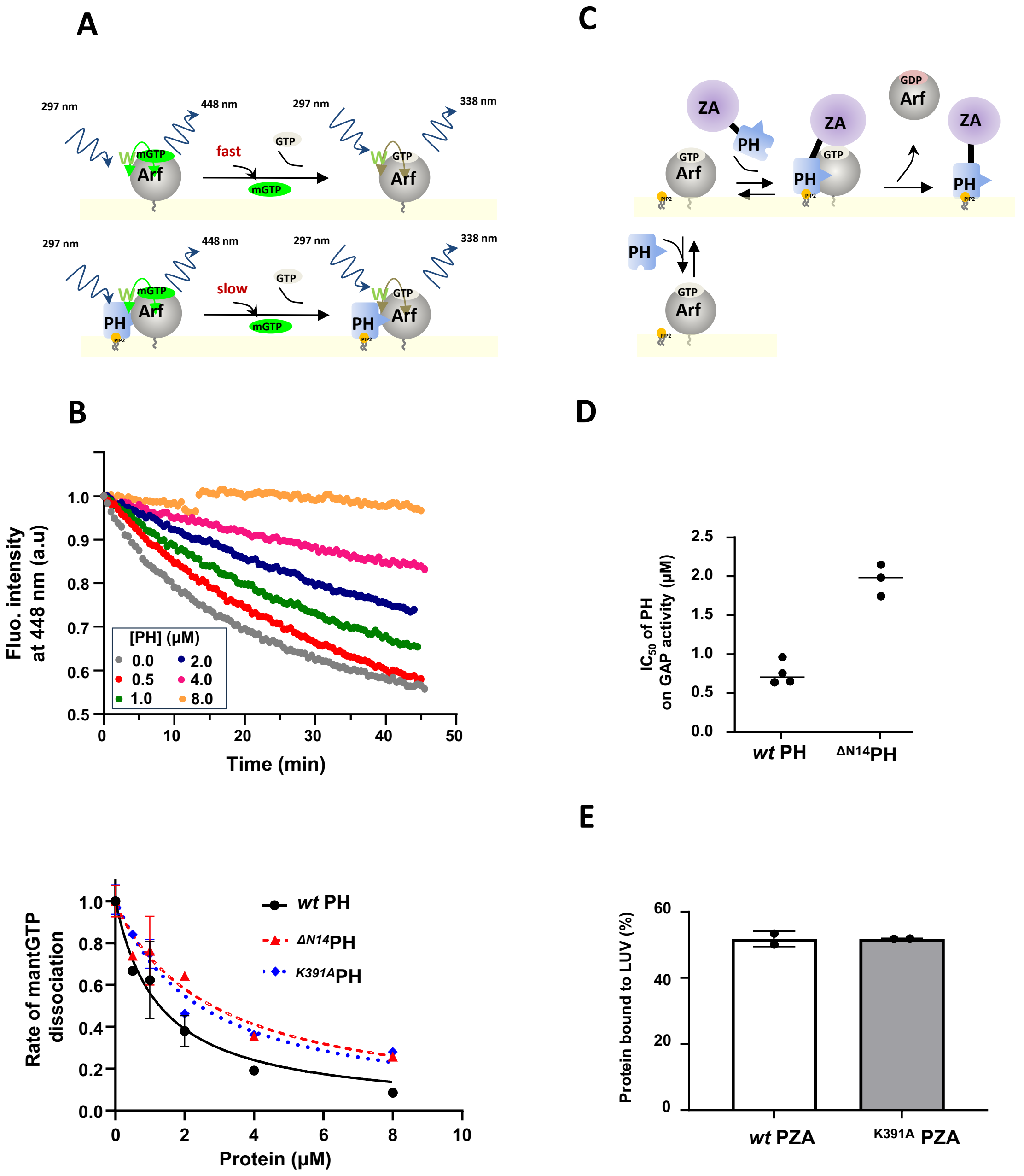

Fig. SI8. (A) Principle of FRET based assay used to measure K_d of PH for Arf1. MyrArf1 was loaded with the nucleotide analog mant-GTP, and the concentration of mant-GTP -bound Arf was

followed by Fluorescence Resonance Energy Transfer (FRET) from myrArf1 tryptophan to mant-GTP. Then, *wt* PH or $^{\Delta N14}$PH was titrated into the reaction containing an excess of GTP. Here, a decrease in FRET indicates mant-GTP dissociation. If the dissociation rate is slower for myrArf1·GTP in complex with the PH domain than for uncomplexed myrArf1·GTP, the concentration of PH domain reducing the rate to ½ the maximum reduction in rate is then equal to the dissociation constant (K_d). **(B) top:** Example of kinetic of FRET intensity measured at increasing concentration of PH domain. **bottom:** Dissociation rates of mantGTP as a function of PH domain concentration for *wt* PH (black circle), $^{\Delta N14}$PH (red triangle) and ^{K391A}PH (blue diamond). The dissociation rates are normalized to the rate measured in the absence of PH domain (0.05 min^{-1}). **(C)** Principle of sequestration from GAP assay used to measure K_d of PH for Arf1. **(D)** Concentration of PH domain necessary to reduce by half GAP activity (IC50) in reactions containing $1 \cdot 10^{-9}$ M myrArf1·GTP (< K_m for *wt* PZA ($1 \cdot 10^{-6}$ M)). Under these conditions, half-maximal inhibition occurs at approximately the K_d. IC50 values (the concentration of PH domain required to reduce GTP hydrolysis by ½ in 3 min) from each independent experiment are shown. Error bars represent standard deviation. ****, $p < 0.0001$ via one-way ANOVA with repeated measures (and mixed effects) and Dunnett's multiple comparisons test against WT. **(E)** Fraction of *wt* PZA or ^{Lys391Ala}PZA bound to $PI(4,5)P_2$ containing LUV measured using a sucrose-loaded vesicle centrifugation assay ([lipid] = 0.5 mM, [protein] = 1 μM), $PI(4,5)P_2$ = 5 mol%. Data are expressed as mean values ± SD (2 repeats).

FIG. SI9

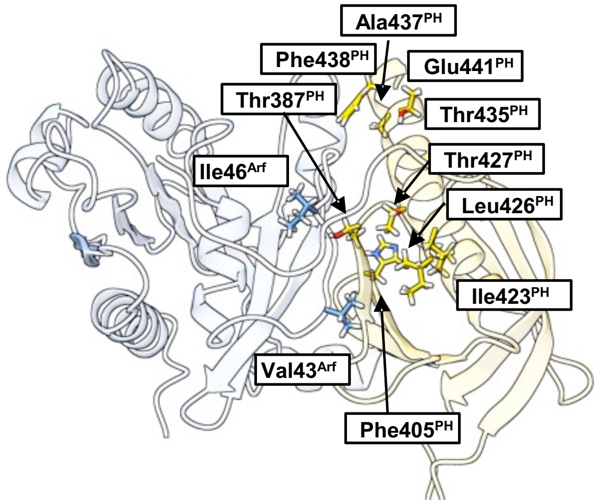

Fig. SI9. Position of mutated residues on myrArf (blue) and ASAP1 PH (gold) tested in this study.

	C50 (in M)	
**Protein**	**+ PI(4,5)P_2**	**- PI(4,5)P_2**
wt PZA	$3 \cdot 10^{-11} \pm 1.5.10^{-11}$ (7)	$1 \cdot 10^{-6} \pm 1.6 \cdot 10^{-6}$ (3)
$^{\Delta N14}$ PZA	$3 \cdot 10^{-9} \pm 1.8.10^{-9}$ (3)	ND
PHdZA	$3 \cdot 10^{-6} \pm 1.7 \cdot 10^{-6}$ (3)	$>1 \cdot 10^{-3}$ (3)
ZA	$> 0.12 \cdot 10^{-3}$ (4)	$> 0.12 \cdot 10^{-3}$ (3)
wt PH + ZA	$4.1 \cdot 10^{-6} \pm 0.3 \cdot 10^{-6}$ (4)	ND
$^{\Delta N14}$PH + ZA	$2 \cdot 10^{-4} \pm 1.7 \cdot 10^{-4}$ (4)	ND

TABLE SI1

Table SI1. Comparison of GAP activity using Arf1 as substrate in membranes with or without $PI(4,5)P_2$. The amount of GAP (in M) required to achieve 50% conversion of [α^{32}P]GTP to [α^{32}P]GDP in 3 min (C_{50}) was estimated and is inversely proportional to enzymatic power. Data are expressed as mean values ± SD with the number of repeats indicated between parentheses.

TABLE SI2

Ambiguous interaction restraints used in the structure calculations of Clusters$^{\beta}{}_5/^{\beta}{}_7$

Protein	Active Residues	Passive Residues
myrArf1	20, 40, 47, 49, 52, 53, 77, 79, 84	51, 55
ASAP1 PH	387, 388, 389, 390, 408, 423, 434, 435, 437	none

Ambiguous interaction restraints used in the structure calculations of Clusters$^{\beta}{}_2/^{\beta}{}_3$

Protein	Active Residues	Passive Residues
myrArf1	20, 40, 47, 49, 52, 53, 77, 79, 84	51, 55
ASAP1 PH	368, 370	364, 371

Unambiguous Distance restraints from PRE measurements used in the structure calculation of Clusters$^{\beta}{}_5/^{\beta}{}_7$

Residue	Sγ atom	Distance (Å)
Ile403	K38C	1.8 - 16

Thr408	K38C	1.8 - 16
Ala394	K38C	1.8 - 16

Unambiguous Distance restraints from PRE measurements used in the structure calculation of Clusters$^{\beta_2/\beta_3}$

Residue	Sγ atom	Distance (Å)
Ala363	K38C	1.8 - 16
Ile368	K38C	1.8 - 16
Thr370	K38C	1.8 - 16

TABLE SI3

myrArf1 protein	GAP assays	
	C_{50} (nM)	Fold change
**WT**	0.036 ± 0.014 (7)	1
**E41A**	0.11 ± 0.02 (3)	3.2
**I46A**	48 ± 2 (3)	1337
**V43A**	0.25 ± 0.04 (4)	6.9
**E54A**	2.57 ± 0.32 (3)	72

Table SI3. Comparison of GAP activity for myrArf1 mutants measured in LUV containing 5 mol% of PI(4,5)P_2. The amount of GAP (in nM) required to achieve 50% conversion of [α^{32}P]GTP to [α^{32}P]GDP in 3 min (C_{50}) was estimated. Data are expressed as mean values ± SD with the number of repeats indicated between parentheses.

TABLE SI4

ASAP1 PZA	GAP assays	
	C_{50} (nM)	Fold change
**WT**	0.036 ± 0.014 (7)	1
**T387L**	0.021 ± 0.005 (4)	0.6
**K391A**	3.58 ± 1.055 (4)	99.8
**H405E**	0.154 ± 0.069 (6)	4.3
**F438A**	0.564 ± 0.28 (4)	15.7

**E441A**	0.023 ± 0.005 (4)	1.2
**E441R**	0.045 ± 0.008 (4)	0.6

Table SI4. Comparison of GAP activity for PZA mutants measured in LUV containing 5 mol% of $PI(4,5)P_2$. The amount of GAP (in nM) required to achieve 50% conversion of [α^{32}P]GTP to [α^{32}P]GDP in 3 min (C_{50}) was estimated. Data are expressed as mean values ± SD with the number of repeats indicated between parentheses.

**ASAP1 PZA**	**GAP assays**	
	C_{50} (nM)	Fold change
**WT***	0.36 ± 0.14 (7)	1
**I423A***	0.756 ± 0.17 (3)	2.1
**L426A***	1.86 ± 0.23 (3)	5.19
**T427A***	0.154 ± 0.1 (3)	0.43
**T435A***	0.129 ± 0.1 (3)	0.36

**A437R***	1.303 ± 0.1 (3)	3.62

TABLE SI5

Table SI5. Comparison of GAP activity for PZA mutants measured in LUVs at 1 mol% PI(4,5)P[2]. The amount of GAP (in nM) required to achieve 50% conversion of [α^{32}P]GTP to [α^{32}P]GDP in 3 min (C_{50}) was estimated. Data are expressed as mean values ± SD with the number of repeats indicated between parentheses. Asterisk indicated determination was made with 1 mol% PIP2 in the LUVs. All other experiments with LUVs included 5 mol% PIP2.

KINETIC MODELING

“In Trans” Reaction Network

Our model reaction network for the in trans experiments tracks the concentrations of 18 species in total. The 5 fundamental species are PH, ZA, Arf·GTP, PS, and PIP_2. Arf complexes formed through various bimolecular association reactions are Arf·GTP, PH• Arf·GTP, PH• Arf·GTP •ZA. Since the PH domain can bind to both PS and PIP_2, we also have distinct PS•PH, PIP_2•PH and PS•PIP_2•PH species. We therefore also must track the 6 corresponding variations of the two PH•Arf complexes. Finally, there is the product Arf·GDP formed through GTP hydrolysis. The list of reactions, along with corresponding rate constants, is:

1. $\mathrm{PIP_2 + PH \rightleftharpoons PIP_2 \bullet PH} : k_{on/off}^{\mathrm{PH+PIP}}$
2. $\mathrm{PS + PH \rightleftharpoons PS \bullet PH} : k_{on/off}^{\mathrm{PH+PS}}$
3. $\mathrm{PIP_2 + PS \bullet PH \rightleftharpoons PS \bullet PIP_2 \bullet PH} : k_{on/off}^{\mathrm{PH+PIP}}$ (2D)
4. $\mathrm{PS + PIP_2 \bullet PH \rightleftharpoons PS \bullet PIP_2 \bullet PH} : k_{on/off}^{\mathrm{PH+PS}}$ (2D)
5. $\mathrm{PIP_2 + PH \bullet ArfGTP \rightleftharpoons PIP_2 \bullet PH \bullet ArfGTP} : k_{on/off}^{\mathrm{PH+PIP}}$ (2D)
6. $\mathrm{PS + PH \bullet ArfGTP \rightleftharpoons PS \bullet PH \bullet ArfGTP} : k_{on/off}^{\mathrm{PH+PS}}$ (2D)
7. $\mathrm{PIP_2 + PS \bullet PH \bullet ArfGTP \rightleftharpoons PS \bullet PIP_2 \bullet PH \bullet ArfGTP} : k_{on/off}^{\mathrm{PH+PIP}}$ (2D)
8. $\mathrm{PS + PIP_2 \bullet PH \bullet ArfGTP \rightleftharpoons PS \bullet PIP_2 \bullet PH \bullet ArfGTP} : k_{on/off}^{\mathrm{PH+PS}}$ (2D)
9. $\mathrm{PIP_2 + PH \bullet ArfGTP \bullet ZA \rightleftharpoons PIP_2 \bullet PH \bullet ArfGTP \bullet ZA} : k_{on/off}^{\mathrm{PH+PIP}}$ (2D)
10. $\mathrm{PS + PH \bullet ArfGTP \bullet ZA \rightleftharpoons PS \bullet PH \bullet ArfGTP \bullet ZA} : k_{on/off}^{\mathrm{PH+PS}}$ (2D)
11. $\mathrm{PIP_2 + PS \bullet PH \bullet ArfGTP \bullet ZA \rightleftharpoons PS \bullet PIP_2 \bullet PH \bullet ArfGTP \bullet ZA} : k_{on/off}^{\mathrm{PH+PIP}}$ (2D)
12. $\mathrm{PS + PIP_2 \bullet PH \bullet ArfGTP \bullet ZA \rightleftharpoons PS \bullet PIP_2 \bullet PH \bullet ArfGTP \bullet ZA} : k_{on/off}^{\mathrm{PH+PS}}$ (2D)
13. $\mathrm{ArfGTP + PH \rightleftharpoons PH \bullet ArfGTP} : k_{on/off}^{\mathrm{Arf+PH}}$
14. $\mathrm{ArfGTP + PS \bullet PH \rightleftharpoons PS \bullet PH \bullet ArfGTP} : k_{on/off}^{\mathrm{Arf+PH}}$ (2D)

15. ArfGTP + PIP_2 • PH ⇌ PIP_2 • PH • ArfGTP : $k_{on/off}^{\text{Arf+PH}}$ (2D)

16. ArfGTP + PS • PIP_2 • PH ⇌ PS • PIP_2 • PH • ArfGTP : $k_{on/off}^{\text{Arf+PH}}$ (2D)

17. ArfGTP • ZA + PH ⇌ PH • ArfGTP • ZA : $k_{on/off}^{\text{Arf+PH}}$

18. ArfGTP • ZA + PS • PH ⇌ PS • PH • ArfGTP • ZA : $k_{on/off}^{\text{Arf+PH}}$ (2D)

19. ArfGTP • ZA + PIP_2 • PH ⇌ PIP_2 • PH • ArfGTP • ZA : $k_{on/off}^{\text{Arf+PH}}$ (2D)

20. ArfGTP • ZA + PS • PIP_2 • PH ⇌ PS • PIP_2 • PH • ArfGTP • ZA : $k_{on/off}^{\text{Arf+PH}}$ (2D)

21. ArfGTP + ZA ⇌ ArfGTP • ZA : $k_{on/off}^{\text{Arf+ZA}}$

22. PH • ArfGTP + ZA ⇌ PH • ArfGTP • ZA : $k_{on/off}^{\text{Arf+ZA}}$

23. PS • PH • ArfGTP + ZA ⇌ PS • PH • ArfGTP • ZA : $k_{on/off}^{\text{Arf+ZA}}$

24. PIP_2 • PH • ArfGTP + ZA ⇌ PIP_2 • PH • ArfGTP • ZA : $k_{on/off}^{\text{Arf+ZA}}$

25. PS • PIP_2 • PH • ArfGTP + ZA ⇌ PS • PIP_2 • PH • ArfGTP • ZA : $k_{on/off}^{\text{Arf+ZA}}$

26. ArfGTP • ZA → ArfGDP + ZA : $k_{cat}^{\text{Arf.ZA}}$

27. PH • ArfGTP • ZA → ArfGDP + PH + ZA : $k_{cat}^{\text{PH.Arf.ZA}}$

28. PS • PH • ArfGTP • ZA → ArfGDP + PS • PH + ZA : $k_{cat}^{\text{PH.Arf.ZA}}$

29. PIP_2 • PH • ArfGTP • ZA → ArfGDP + PIP_2 • PH + ZA : $k_{cat}^{\text{PH.Arf.ZA}}$

30. PS • PIP_2 • PH • ArfGTP • ZA → ArfGDP + PS • PIP_2 • PH + ZA : $k_{cat}^{\text{PH.Arf.ZA}}$

Arf·GTP is assumed to reside entirely on the membrane due to its N-terminal myristoylation. Rates followed by "(2D)" indicate reactions which occur on the two-dimensional membrane surface. The association rates for 2D reactions (dimensions $area^{-1}time^{-1}$) are related to their 3D counterparts (dimensions $volume^{-1}time^{-1}$) by dividing by a nanoscopic length scale h which phenomenologically accounts for thermodynamic effects of surface binding. h is typically comparable to the molecular

length scale, i.e., of order nanometers [5]. Since in our ODE system we track all species based on volume concentration, the effective macroscopic association rates (dimensions *volume*$^{-1}$*time*$^{-1}$) for 2D reactions used in these equations are multiplied by a unitless *dimensionality factor* $\mathrm{DF} = V/Ah$, where V is the solution volume and A is the total membrane area. Critically, the k_{cat} rate is allowed to change based on the presence of PH in complex with Arf. A simplifying assumption made in this model is that the rates of association/dissociation for PH to Arf are independent of whether or not lipids are bound. Additionally, the association of PH to the membrane via $\mathrm{PIP_2}$ and PS binding is modeled in both cases as a single bimolecular association reaction.

Tandem network

The tandem PZA reaction network is qualitatively different from the "in trans" system due to the presence of the linker between the PH and ZA subunits. After the binding of either the PH or ZA subunit to Arf, the subsequent binding of the other subunit becomes a first-order reaction rather than second-order. We introduce rates for these "loop-closure" reactions in the following way:

The dissociation constants for the individual subunits binding to Arf can be expressed as

$$K_d^{\mathrm{Arf+ZA}} = \frac{k_{off}^{\mathrm{Arf+ZA}}}{k_{on}^{\mathrm{Arf+ZA}}} = c_0 e^{\,\Delta G_{\mathrm{ZA}}/k_{\mathrm{B}}T} \qquad K_d^{\mathrm{Arf+PH}} = \frac{k_{off}^{\mathrm{Arf+PH}}}{k_{on}^{\mathrm{Arf+PH}}} = c_0 e^{\,\Delta G_{\mathrm{PH}}/k_{\mathrm{B}}T}$$

where the $k_{on/off}$ rates are the same as those in the reactions above, $\Delta G_{\mathrm{ZA/PH}}$ is the change in Gibbs free energy upon ZA/PH binding, and c_0 is the standard state concentration 1 M. The affinity of PZA for Arf can be expressed as

$$K_d^{\mathrm{Arf+PZA}} = c_0 e^{\,(\Delta G_{\mathrm{ZA}} + \Delta G_{\mathrm{PH}} + \Delta G_{\mathrm{coop}})/k_{\mathrm{B}}T}$$

where ΔG_{coop} is a cooperative contribution to the free energy of binding for PH and ZA to Arf due to the linker connecting them. Substituting the expressions above for $\Delta G_{\mathrm{ZA/PH}}$ in terms of rates, we arrive at

$$K_d^{\mathrm{Arf+PZA}} = \frac{k_{off}^{\mathrm{Arf+ZA}}\, k_{off}^{\mathrm{Arf+PH}}}{c_0 k_{on}^{\mathrm{Arf+ZA}}\, k_{on}^{\mathrm{Arf+PH}}} e^{\Delta G_{\mathrm{coop}}/k_{\mathrm{B}}T}.$$

Considering the case where PH binds first and rearranging this expression we find,

$$K_d^{\text{Arf+PZA}} = \frac{k_{off}^{\text{Arf+PH}}}{k_{on}^{\text{Arf+PH}}} \frac{k_{off}^{\text{Arf+ZA}}}{c_0 k_{on}^{\text{Arf+ZA}} \, e^{-\Delta G_{\text{coop}}/k_{\text{B}}T}} \, .$$

So, assuming that the off-rate $k_{off}^{\text{Arf+ZA}}$ is unchanged, we can straightforwardly read off the first-order binding rate for ZA to "close the loop" to Arf after PH is already bound: $k_{close}^{\text{Arf+ZA}} = c_0 k_{on}^{\text{Arf+ZA}} \, e^{-\Delta G_{\text{coop}}/k_{\text{B}}T}$. Rearranging the terms produces the analogous expression for PH loop-closure. The reactions which use these rates are highlighted with an asterisk (*) in the list below. That the off-rate remains the same is a reasonable simplifying assumption that we make here; however, this can be relaxed at the cost of introducing a new parameter determining what fraction of $c_0 \, e^{-\Delta G_{\text{coop}}/k_{\text{B}}T}$ is attributed to the on vs. off rate.

Although these reactions all have the wild type tandem PZA unit, we still use the notation of PH and ZA for PZA in complex with Arf in order to track which subunit is bound. For example, the species PH • ArfGTP represents PZA in complex with Arf wherein the PH domain is bound to Arf, but the ZA domain is not. In the following list, the notation ZA_{PIP}, ZA_{PS}, and $\text{ZA}_{\text{PS.PIP}}$ are used to track the PH lipid binding state for species in which only the ZA domain is bound to Arf.

1. $\text{PIP}_2 + \text{PZA} \rightleftharpoons \text{PIP}_2 \bullet \text{PZA} \; : \; k_{on/off}^{\text{PH+PIP}}$
2. $\text{PS} + \text{PZA} \rightleftharpoons \text{PS} \bullet \text{PZA} \; : \; k_{on/off}^{\text{PH+PS}}$
3. $\text{PIP}_2 + \text{PS} \bullet \text{PZA} \rightleftharpoons \text{PS} \bullet \text{PIP}_2 \bullet \text{PZA} \; : \; k_{on/off}^{\text{PH+PIP}}$ (2D)
4. $\text{PS} + \text{PIP}_2 \bullet \text{PZA} \rightleftharpoons \text{PS} \bullet \text{PIP}_2 \bullet \text{PZA} \; : \; k_{on/off}^{\text{PH+PS}}$ (2D)
5. $\text{PIP}_2 + \text{PH} \bullet \text{ArfGTP} \rightleftharpoons \text{PIP}_2 \bullet \text{PH} \bullet \text{ArfGTP} \; : \; k_{on/off}^{\text{PH+PIP}}$ (2D)
6. $\text{PS} + \text{PH} \bullet \text{ArfGTP} \rightleftharpoons \text{PS} \bullet \text{PH} \bullet \text{ArfGTP} \; : \; k_{on/off}^{\text{PH+PS}}$ (2D)
7. $\text{PIP}_2 + \text{PS} \bullet \text{PH} \bullet \text{ArfGTP} \rightleftharpoons \text{PS} \bullet \text{PIP}_2 \bullet \text{PH} \bullet \text{ArfGTP} \; : \; k_{on/off}^{\text{PH+PIP}}$ (2D)
8. $\text{PS} + \text{PIP}_2 \bullet \text{PH} \bullet \text{ArfGTP} \rightleftharpoons \text{PS} \bullet \text{PIP}_2 \bullet \text{PH} \bullet \text{ArfGTP} \; : \; k_{on/off}^{\text{PH+PS}}$ (2D)
9. $\text{PIP}_2 + \text{PH} \bullet \text{ArfGTP} \bullet \text{ZA} \rightleftharpoons \text{PIP}_2 \bullet \text{PH} \bullet \text{ArfGTP} \bullet \text{ZA} \; : \; k_{on/off}^{\text{PH+PIP}}$ (2D)
10. $\text{PS} + \text{PH} \bullet \text{ArfGTP} \bullet \text{ZA} \rightleftharpoons \text{PS} \bullet \text{PH} \bullet \text{ArfGTP} \bullet \text{ZA} \; : \; k_{on/off}^{\text{PH+PS}}$ (2D)

11. $PIP_2 + PS \bullet PH \bullet ArfGTP \bullet ZA \rightleftharpoons PS \bullet PIP_2 \bullet PH \bullet ArfGTP \bullet ZA$: $k_{on/off}^{\mathrm{PH+PIP}}$ (2D)

12. $PS + PIP_2 \bullet PH \bullet ArfGTP \bullet ZA \rightleftharpoons PS \bullet PIP_2 \bullet PH \bullet ArfGTP \bullet ZA$: $k_{on/off}^{\mathrm{PH+PS}}$ (2D)

13. $PIP_2 + ArfGTP \bullet ZA \rightleftharpoons PH \bullet ArfGTP \bullet ZA_{PIP}$: $k_{on/off}^{\mathrm{PH+PIP}}$ (2D)

14. $PS + ArfGTP \bullet ZA \rightleftharpoons ArfGTP \bullet ZA_{PS}$: $k_{on/off}^{\mathrm{PH+PS}}$ (2D)

15. $PIP_2 + ArfGTP \bullet ZA_{PS} \rightleftharpoons ArfGTP \bullet ZA_{PS,PIP}$: $k_{on/off}^{\mathrm{PH+PIP}}$ (2D)

16. $PS + ArfGTP \bullet ZA \rightleftharpoons PH \bullet ArfGTP \bullet ZA_{PS,PIP}$: $k_{on/off}^{\mathrm{PH+PS}}$ (2D)

17. $ArfGTP + PZA \rightleftharpoons PH \bullet ArfGTP$: $k_{on/off}^{\mathrm{Arf+PH}}$

18. $ArfGTP + PS \bullet PZA \rightleftharpoons PS \bullet PH \bullet ArfGTP$: $k_{on/off}^{\mathrm{Arf+PH}}$ (2D)

19. $ArfGTP + PIP_2 \bullet PZA \rightleftharpoons PIP_2 \bullet PH \bullet ArfGTP$: $k_{on/off}^{\mathrm{Arf+PH}}$ (2D)

20. $ArfGTP + PS \bullet PIP_2 \bullet PH \rightleftharpoons PS \bullet PIP_2 \bullet PH \bullet ArfGTP$: $k_{on/off}^{\mathrm{Arf+PH}}$ (2D)

21. $ArfGTP + PZA \rightleftharpoons ArfGTP \bullet ZA$: $k_{on/off}^{\mathrm{Arf+ZA}}$

22. $ArfGTP + PIP_2 \bullet PZA \rightleftharpoons ArfGTP \bullet ZA_{PIP}$: $k_{on/off}^{\mathrm{Arf+ZA}}$ (2D)

23. $ArfGTP + PS \bullet PZA \rightleftharpoons ArfGTP \bullet ZA_{PS}$: $k_{on/off}^{\mathrm{Arf+ZA}}$ (2D)

24. $ArfGTP + PS \bullet PIP_2 \bullet PZA \rightleftharpoons ArfGTP \bullet ZA_{PS,PIP}$: $k_{on/off}^{\mathrm{Arf+ZA}}$ (2D)

25. $PH \bullet ArfGTP \rightleftharpoons PH \bullet ArfGTP \bullet ZA$: $k_{close/off}^{\mathrm{Arf+ZA}}$ *

26. $PIP_2 \bullet PH \bullet ArfGTP \rightleftharpoons PIP_2 \bullet PH \bullet ArfGTP \bullet ZA$: $k_{close/off}^{\mathrm{Arf+ZA}}$ *

27. $PS \bullet PH \bullet ArfGTP \rightleftharpoons PS \bullet PH \bullet ArfGTP \bullet ZA$: $k_{close/off}^{\mathrm{Arf+ZA}}$ *

28. $PS \bullet PIP_2 \bullet PH \bullet ArfGTP \rightleftharpoons PS \bullet PIP_2 \bullet PH \bullet ArfGTP \bullet ZA$: $k_{close/off}^{\mathrm{Arf+ZA}}$ *

29. $ArfGTP \bullet ZA \rightleftharpoons PH \bullet ArfGTP \bullet ZA$: $k_{close/off}^{\mathrm{Arf+PH}}$ *

30. $\mathrm{ArfGTP \bullet ZA_{PIP} \rightleftharpoons PIP_2 \bullet PH \bullet ArfGTP \bullet ZA}$: $k_{close/off}^{\mathrm{Arf+PH}}$ *

31. $\mathrm{ArfGTP \bullet ZA_{PS} \rightleftharpoons PS \bullet PH \bullet ArfGTP \bullet ZA}$: $k_{close/off}^{\mathrm{Arf+PH}}$ *

32. $\mathrm{ArfGTP \bullet ZA_{PS,PIP} \rightleftharpoons PS \bullet PIP_2 \bullet PH \bullet ArfGTP \bullet ZA}$: $k_{close/off}^{\mathrm{Arf+PH}}$ *

33. $\mathrm{ArfGTP \bullet ZA \rightarrow ArfGDP + ZA}$: $k_{cat}^{\mathrm{Arf.ZA}}$

34. $\mathrm{ArfGTP \bullet ZA_{PIP} \rightarrow ArfGDP + PIP_2 \bullet PZA}$: $k_{cat}^{\mathrm{Arf.ZA}}$

35. $\mathrm{ArfGTP \bullet ZA_{PS} \rightarrow ArfGDP + PS \bullet PZA}$: $k_{cat}^{\mathrm{Arf.ZA}}$

36. $\mathrm{ArfGTP \bullet ZA_{PS,PIP} \rightarrow ArfGDP + PS \bullet PIP_2 \bullet PZA}$: $k_{cat}^{\mathrm{Arf.ZA}}$

37. $\mathrm{PH \bullet ArfGTP \bullet ZA \rightarrow ArfGDP + PH + ZA}$: $k_{cat}^{\mathrm{PZA.Arf}}$

38. $\mathrm{PS \bullet PH \bullet ArfGTP \bullet ZA \rightarrow ArfGDP + PS \bullet PH + ZA}$: $k_{cat}^{\mathrm{PZA.Arf}}$

39. $\mathrm{PIP_2 \bullet PH \bullet ArfGTP \bullet ZA \rightarrow ArfGDP + PIP_2 \bullet PH + ZA}$: $k_{cat}^{\mathrm{PZA.Arf}}$

40. $\mathrm{PS \bullet PIP_2 \bullet PH \bullet ArfGTP \bullet ZA \rightarrow ArfGDP + PS \bullet PIP_2 \bullet PH + ZA}$: $k_{cat}^{\mathrm{PZA.Arf}}$

Two further assumptions have been made such that the reaction rates for this system are determined entirely in terms of the rates for the in trans reactions and the new parameter ΔG_{coop}. First, as PS and PIP_2 binding occur on the PH domain, the rates for membrane association of PZA are taken equal to those for PH alone. Second, the catalytic rate $k_{cat}^{\mathrm{PZA.Arf}}$ is taken equal to the previously measured experimental value of approximately 56 s^{-1} [7].

The simplified PHdZA model (green curves in Fig. SI10A) uses the same reaction network as PZA with modified kinetic parameters: PHd binding to Arf is disallowed, as PHd has negligible affinity for Arf, and therefore k_{cat} is assumed to be always equal to $k_{cat}^{\mathrm{Arf.ZA}}$.

Parameter Optimization based on Experimental Data

FIG. SI10

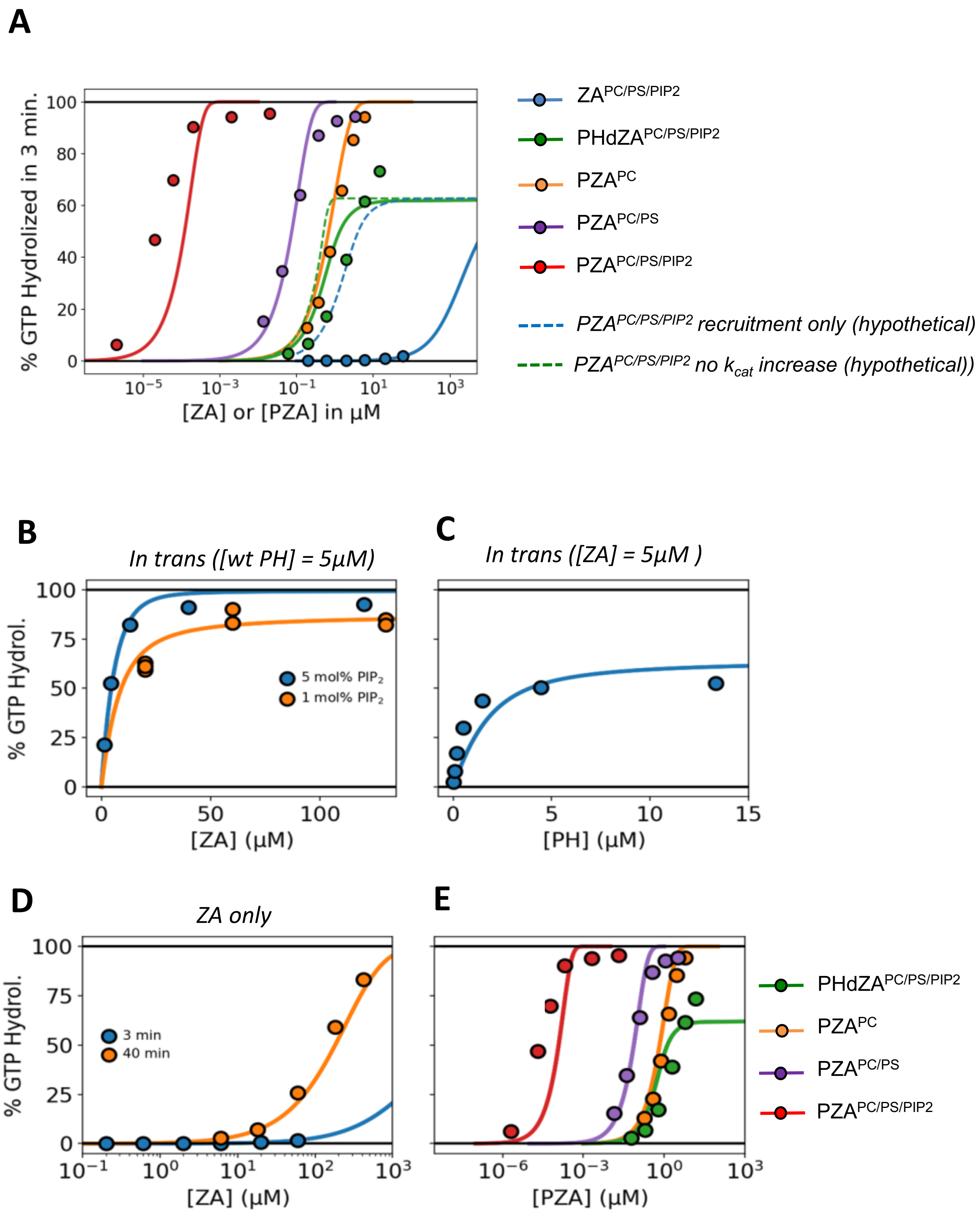
A
% GTP Hydrolized in 3 min.
[ZA] or [PZA] in µM
ZA^PC/PS/PIP2
PHdZA^PC/PS/PIP2
PZA^PC
PZA^PC/PS
PZA^PC/PS/PIP2
PZA^PC/PS/PIP2 recruitment only (hypothetical)
PZA^PC/PS/PIP2 no k_cat increase (hypothetical))
B
In trans ([wt PH] = 5µM)
% GTP Hydrol.
5 mol% PIP2
1 mol% PIP2
[ZA] (µM)
C
In trans ([ZA] = 5µM)
[PH] (µM)
D
ZA only
% GTP Hydrol.
3 min
40 min
[ZA] (µM)
E
[PZA] (µM)
PHdZA^PC/PS/PIP2
PZA^PC
PZA^PC/PS
PZA^PC/PS/PIP2

Fig. SI10. Kinetic modeling of GAP activity. (A) Fraction of GTP hydrolyzed in 3 minutes by either ZA or PZA. From right to left, solid curves are for ZA (blue), PHdZA with PI(4,5)P_2 and PS (green), PZA (orange), PZA with PS (purple), and PZA with both PS and PI(4,5)P_2 (red). Dashed curves are hypothetical PZA in which the sole PH function is substrate binding (blue dashed) and hypothetical PZA in which PH does not induce allosteric enhancement in k_{cat} (green dashed). Points represent experimental data and lines are predictions from optimized kinetic ODE model. In all cases where PI(4,5)P_2 is present its concentration is 5 mol%, and similarly 15 mol% for PS. **(B–E)** Plots of experimental data (circles, triangles) used to optimize the kinetic model, along with model predictions (solid curves) for the final optimal parameter set. Initial Arf·GTP concentration is 1μM in all cases. Unless indicated otherwise, PI(4,5)P_2 concentration is 5 mol%. Reaction time is 3 minutes except where otherwise indicated.

The model parameters are constrained by experimental GTP hydrolysis rates as determined in several different conditions for both in trans and tandem PZA, as well as PHdZA. For the in trans systems, these include data for fraction of GTP hydrolyzed in 3 minutes with a fixed concentration of PH and varying amount of ZA (Fig. SI10B), fixed concentration of ZA and varying PH (Fig. SI10C), and ZA alone with no PH present (Fig. SI10D). For tandem PZA, we use fraction of GTP hydrolyzed in the absence of PIP_2 and PS, with 15 mol% PS, and with 15 mol% PS and 5 mol% PIP_2 present (Fig. SI10E).

Fig. SI10A also presents two hypothetical scenarios generated using the optimal model parameters, shown as dashed curves. The blue dashed curve presents a tandem PZA scenario in which the sole function of the PH domain is substrate binding and does not bind to lipids nor induce allosteric changes to the catalytic rate. The green dashed curve adds lipid binding (PS and PIP_2) to the situation just described. Together with the solid green curve (approximate model for PHdZA), these demonstrate that while dimensional reduction and substrate binding are each individually able to produce large enhancements in GAP activity compared to ZA alone, their

combined effects (dashed green curve) are limited to roughly 4 order-of-magnitude enhancement. In addition, halving the affinity of the PH domain for Arf (simulating the difference in affinity between $^{\Delta N14}$PZA and *wt* PZA) in a model with recruitment and binding, but without conformational change in Arf resulted in a near negligible impact on overall GAP activity by PZA. These results are consistent with the critical role of the intrinsically disordered region of the PH domain for GAP activity.

Our model parameters were optimized by minimizing the χ^2 residual between the simulation results and the observational data points, while imposing certain restrictions on the model parameters. In addition to the parameter limits given in Table SI7, the following constraints were imposed on dissociation constants $K_\mathrm{d} = k_\mathrm{off}/k_\mathrm{on}$ based on experimental measurements: $K_\mathrm{d}^\mathrm{PH+Arf} \geq 10\mu\mathrm{M}$, $K_\mathrm{d}^\mathrm{ZA+Arf} \geq 100\mu\mathrm{M}$, and $K_\mathrm{d}^\mathrm{ZA+Arf} > K_\mathrm{d}^\mathrm{PH+Arf}$. Additionally, the following constraint was imposed on rate constants to avoid pathological results: $k_{cat}^\mathrm{PH.Arf.ZA} \geq k_{cat}^\mathrm{Arf.ZA}$. These restrictions, along with parameters which are held constant during fitting, are summarized in Table SI6.

In order to find optimal parameter combinations in the high-dimensional space, we performed stochastic global optimization via a genetic algorithm implemented in the Julia programming language using packages from the SciML ecosystem [1–4]. An initial population of 15000 sets of parameters (individual candidates in the evolutionary algorithm) is sampled uniformly in log-space from the allowable parameter ranges (Table SI7) and respecting the imposed constraints (Table SI6). The genetic algorithm then proceeds by iterating the following steps for 10 generations:

1. Generate an offspring population via crossover and mutation:
    a. Adjacent candidate pairs have 50% probability of swapping parameters via 2-point crossover.
    b. Each individual parameter within a candidate then has a 75% chance of being uniformly scaled by up to 50% in either direction (within the prescribed bounds).

c. Additionally, the 5 best individuals from the previous generation continue on unmodified.

2. Evaluate each candidate's fitness as the χ^2 residual between the numerical ODE result and the data in Fig. SI10B-E.

3. Select the next generation by tournament: 10% of the population is selected at random, and the individual with highest fitness is added to the next generation. This is repeated until the next generation has equal size.

This entire process was repeated several times and the best final set of parameters (lowest χ^2) over all runs was taken as the optimal parameter set; the values are given in Table SI7. The χ^2 value for the optimal parameters is roughly 28% lower than the χ^2 of the next-best local optimum found, distinguishing it rather strongly from other candidate parameter sets. This solution is plotted in Fig. 8 and Fig. SI10, and the parameter uncertainties are presented in Fig. SI11.

The large sample of initial candidate points followed by subsequent generations of stochastic updates allows the algorithm to sample many regions of the parameter space in order to avoid becoming trapped in an initial local minimum of the fitness landscape. However, the stochastic nature of the algorithm, combined with the rough nature of the high-dimensional fitness landscape, does mean that repeated optimization runs generally do not converge to identical sets of optimal parameters. Applying a deterministic minimization algorithm (such as downhill simplex) to the optimal candidate post-GA does not resolve this issue.

**Fixed Parameters / Constraints**	
**Parameter**	**Value / Constraint**
$k_{cat}^{\mathrm{PZA.Arf}}$	56 s^{-1} [7]
$K_{\mathrm{d}}^{\mathrm{Arf+PH}}$	≥ 10 μM
$K_{\mathrm{d}}^{\mathrm{Arf+ZA}}$	≥ 100 μM; ≥ $K_{\mathrm{d}}^{\mathrm{Arf+PH}}$

$k_{cat}^{\mathrm{PH.Arf.ZA}}$	$\geq\ k_{cat}^{\mathrm{Arf.ZA}}$

Table SI6. Kinetic model parameters which are fixed or constrained.

**Variable Fit Parameters**			
**Parameter**	**Units**	**Allowed Range**	**Best Fit**
$k_{on}^{\mathrm{Arf+ZA}}$	$\mu M^{-1}s^{-1}$	10^{-8} — 10	0.0056
$k_{off}^{\mathrm{Arf+ZA}}$	s^{-1}	10^{-3} — 10^{5}	18
$k_{on}^{\mathrm{Arf+PH}}$	$\mu M^{-1}s^{-1}$	10^{-8} — 10	6.5×10^{-7}
$k_{off}^{\mathrm{Arf+PH}}$	s^{-1}	10^{-3} — 10^{5}	0.0012
$k_{cat}^{\mathrm{Arf.ZA}}$	s^{-1}	10^{-6} — 56	0.0055
$k_{cat}^{\mathrm{PH.Arf.ZA}}$	s^{-1}	10^{-6} — 56	5.2
$k_{on}^{\mathrm{PH+PIP}}$	$\mu M^{-1}s^{-1}$	10^{-8} — 10	6.9
$K_{d}^{\mathrm{PH+PIP}}$	μM	1 — 100	23
$k_{on}^{\mathrm{PH+PS}}$	$\mu M^{-1}s^{-1}$	10^{-8} — 10	2.0
$K_{d}^{\mathrm{PH+PS}}$	μM	0.1 — 1000	51
h	nm	1 — 100	1

$\exp(-\Delta G_{\mathrm{coop}} / k_{\mathrm{B}}T)$		10^{-5} — 10^{5}	5800

Table SI7. Variable model parameters with allowable ranges and optimal values.

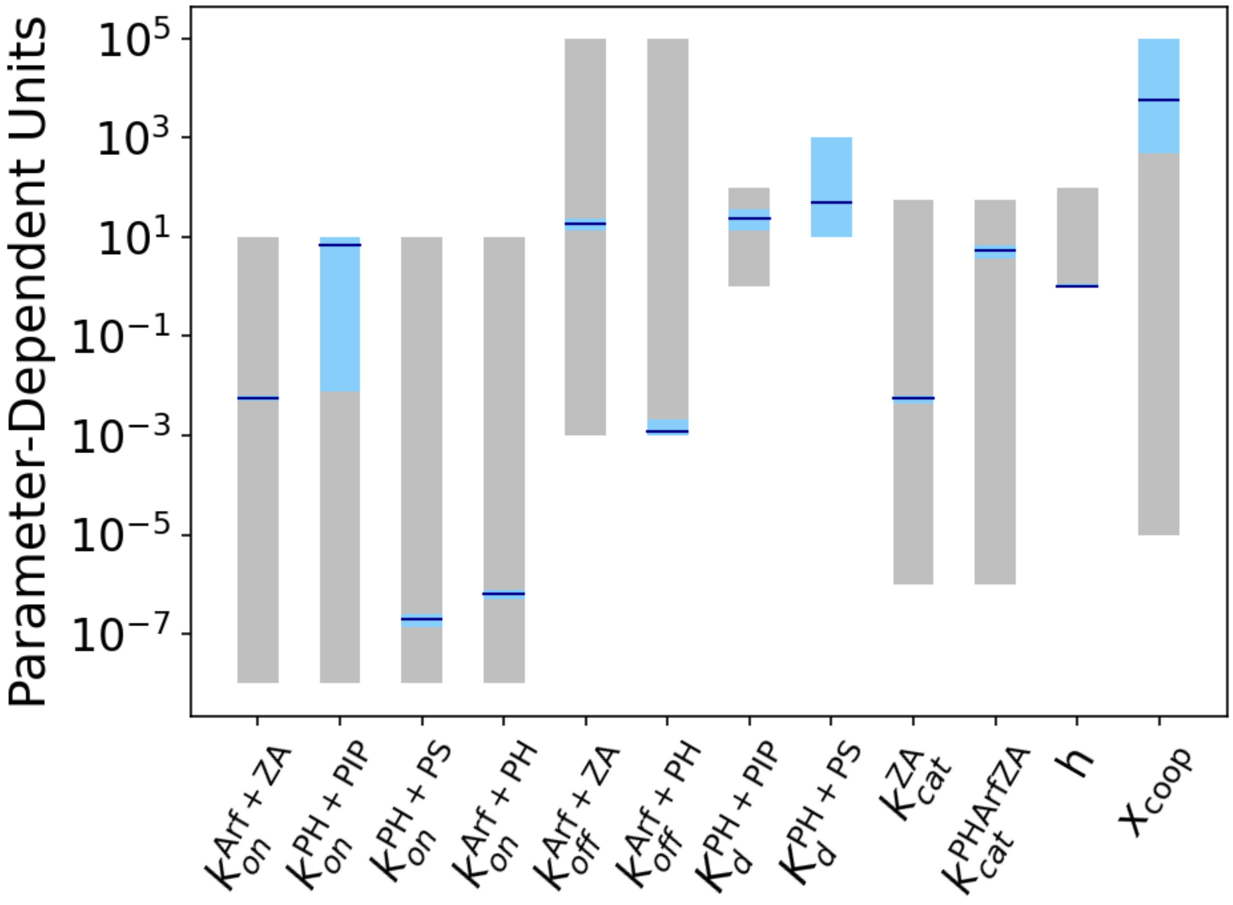

Figure SI11. Parameter values and sensitivities for the optimal solution found by the genetic algorithm. Units for each parameter are given in column 2 of Table SI7. The overall optimal

parameter set (lowest χ^2, values shown in Table SI7) is shown by the dark blue horizontal lines. The light blue shading indicates the range of values for the parameter for which the χ^2 is within 10% of the optimal value. Grey shaded regions indicate allowed parameter ranges during optimization (column 3 of Table SI7). Parameter $x_{\mathrm{coop}} = \exp(-\Delta G_{\mathrm{coop}} / k_{\mathrm{B}}T)$.

Supplementary References

1.Bezanson, J., Edelman, A., Karpinski, S. & Shah, V. B. Julia: A Fresh Approach to Numerical Computing. SIAM Rev. 59, 65–98 (2017).

2.Rackauckas, C. & Nie, Q. DifferentialEquations.jl – A Performant and Feature-Rich Ecosystem for Solving Differential Equations in Julia. Journal of Open Research Software 5, (2017).

3.Art et al. Wildart/Evolutionary.Jl: V0.11.1. (Zenodo, 2022). doi:10.5281/zenodo.5851574.

4.Loman, T. E. et al. Catalyst: Fast and flexible modeling of reaction networks. PLOS Computational Biology 19, e1011530 (2023).

5.Wu, Y., Vendome, J., Shapiro, L., Ben-Shaul, A. & Honig, B. Transforming binding affinities from three dimensions to two with application to cadherin clustering. Nature 475, 510–513 (2011).

6.Jian, X. et al. Molecular Basis for Cooperative Binding of Anionic Phospholipids to the PH Domain of the Arf GAP ASAP1. Structure 23, 1977–1988 (2015).

7.Luo, R. et al. Kinetic analysis of GTP hydrolysis catalysed by the Arf1-GTP–ASAP1 complex. Biochemical Journal 402, 439–447 (2007).
